# A systematic review and quality of reporting checklist for repeatability and reproducibility of radiomic features

**DOI:** 10.1016/j.phro.2021.10.007

**Published:** 2021-11-09

**Authors:** Elisabeth Pfaehler, Ivan Zhovannik, Lise Wei, Ronald Boellaard, Andre Dekker, René Monshouwer, Issam El Naqa, Jan Bussink, Robert Gillies, Leonard Wee, Alberto Traverso

**Affiliations:** a Department of Nuclear Medicine and Molecular Imaging, Medical Imaging Center, University of Groningen, University Medical Center Groningen, Groningen, The Netherlands; b Department of Radiation Oncology, Radboud Institute for Health Sciences, Radboud University Medical Center, Nijmegen, The Netherlands; c Department of Radiation Oncology (MAASTRO), GROW School for Oncology, Maastricht University Medical Centre+, Maastricht, The Netherlands; d Department of Radiation Oncology, University of Michigan, Ann Arbor, MI, USA; e Department of Radiology & Nuclear Medicine, VU University Medical Center, Amsterdam, The Netherlands; f Department of Radiology, Moffitt Cancer Center, Tampa, FL, USA

**Keywords:** Radiomics, Repeatability, Reproducibility, Review

## Abstract

**Purpose:**

Although quantitative image biomarkers (radiomics) show promising value for cancer diagnosis, prognosis, and treatment assessment, these biomarkers still lack reproducibility. In this systematic review, we aimed to assess the progress in radiomics reproducibility and repeatability in the recent years.

**Methods and materials:**

Four hundred fifty-one abstracts were retrieved according to the original PubMed search pattern with the publication dates ranging from 2017/05/01 to 2020/12/01. Each abstract including the keywords was independently screened by four observers. Forty-two full-text articles were selected for further analysis. Patient population data, radiomic feature classes, feature extraction software, image preprocessing, and reproducibility results were extracted from each article. To support the community with a standardized reporting strategy, we propose a specific reporting checklist to evaluate the feasibility to reproduce each study.

**Results:**

Many studies continue to under-report essential reproducibility information: all but one clinical and all but two phantom studies missed to report at least one important item reporting image acquisition. The studies included in this review indicate that all radiomic features are sensitive to image acquisition, reconstruction, tumor segmentation, and interpolation. However, the amount of sensitivity is feature dependent, for instance, textural features were, in general, less robust than statistical features.

**Conclusions:**

Radiomics repeatability, reproducibility, and reporting quality can substantially be improved regarding feature extraction software and settings, image preprocessing and acquisition, cutoff values for stable feature selection. Our proposed radiomics reporting checklist can serve to simplify and improve the reporting and, eventually, guarantee the possibility to fully replicate and validate radiomic studies.

## Highlights

• Main factors impacting feature stability: Image acquisition, reconstruction, tumor segmentation, and interpolation.
• Textural features are less robust than morphological or statistical features.
• A checklist is provided including items that should be reported in a radiomic study.

## 1 Introduction

“Radiomics”, the automated extraction of imaging biomarkers from patients’ scans has gained an increasing interest in the last decade. Several radiomics studies have reported promising results for cancer diagnosis, prognosis, or evaluation of treatment response [1], [2], [3]. Radiomics studies span common volumetric imaging modalities such as Computed Tomography (CT), Positron Emission Tomography (PET), and Magnetic Resonance Imaging (MRI). The numbers of studies investigating applications of radiomics have increased dramatically in the last years. In 2019 alone, 728 studies were indexed in PubMed relating to radiomics studies. However, there remains a translational gap between academic study and clinical utilization [4]. One challenge that makes a clinical implementation of radiomics difficult is the problem of replicating published results. These difficulties are due to the unavailability of input images and software used for computations, poor reporting of study design, and lack of metadata associated with radiomic studies [5]. External validation of radiomic models has been hampered by models trained on small institutional cohorts, prevalence of overfitting and high false positive discovery rates [6]. Additionally, there is a dissonance between the study design methodology and guidelines from TRIPOD (transparent reporting of a multivariable prediction model for individual prognosis or diagnosis) [7] that strongly recommends the validation of prediction models on independent (non-randomly partitioned) datasets and full transparency in reporting.

In a previous review aiming to find a consensus in literature on reproducible and repeatable features, the authors indicated a lack of clear consensus in radiomics methodology. Moreover, the review concluded that deficiencies in reporting of study design, methodology and results were hampering the transparency and reproducibility of radiomic studies [5]. However, the previous effort to identify a subset of features likely to be generally reproducible and repeatable, via a quantitative meta-analysis, was encumbered by two issues identified in the literature: (i) incomplete reporting of radiomic analysis procedure and (ii) heterogeneity in metrics used to report feature repeatability and/or reproducibility. Thus, it was not possible in previous work to perform a conclusive quantitative meta-analysis for every imaging modality (CT, PET, MRI).

Considering the increase in radiomic studies, this work is a revised review about the repeatability and reproducibility of radiomic features, with multiple aims: 1. to identify a set of repeatable/reproducible features for each imaging modality; 2. to verify if consensus has recently emerged regarding a list of major factors impacting on reproducibility/repeatability; 3. to isolate a set of repeatable/reproducible features across different modalities in both human and phantom data; 4. to verify if one of the largest issues identified in previous work, being the poor quality of reporting, has been addressed in new studies. To address aim (4) and support high-quality reporting of radiomic studies, we propose a checklist, which includes all necessary steps to fully reproduce a radiomic study.

## 2 Methods and Materials

### 2.1 Eligibility criteria

Peer-reviewed full-text articles in the English language eligible for this review must have been published between 2017/05/01 to 2020/12/01. One electronic database (PubMed) was used to search for records (see supplementary material for query filter). Only studies investigating radiomic features extracted A) from one of the imaging modalities CT, PET, or MRI and B) from radiologic phantoms or from human persons suffering from at least one primary tumor were eligible for review. Included articles had to report on the repeatability/reproducibility of radiomic features at least oneof the following aspects: image acquisition/reconstruction parameters, effect of image pre-processing such as smoothing, or segmentation method. Studies must report a statistical metric assessing the degree of robustness (such as the Interclass-Correlation Coefficient (ICC)).

### 2.2 Study records

*Selection process:* After the literature search, titles and abstracts were checked for matching the described criteria by four independent observers. Each reviewer voted if an article was eligible for review. In case of disagreement amongst reviewers, a consensus was obtained by joint discussion. EP and IZ reviewed the phantom studies, while AT and LIW reviewed the patient studies.

*Data extraction:* We extracted information about the datasets used for radiomics (e.g. primary tumor type, or phantom details), details about the segmentation method used, details about radiomic feature extraction such as the interpolation method, details about the statistical analysis and the summary of results. Details of the reviewer form are given in Table 1a. DICOM attributes about the important imaging details are summarized in Table 1b for the imaging modalities.

*Creation of the radiomic reporting checklist:* In order to generate a radiomic reporting checklist, we attempted to literally reproduce each step of a study by following the description in each manuscript. The checklist complements the points mentioned by Vallieres et al. [8] and the IBSI reference manual [9], [10] and necessary information to reproduce a patient study (i.e. patient inclusion, statistical analysis). This checklist was also inspired by the QUADAS-2, a tool for the quality assessment of diagnostic accuracy studies [9]. The development of “signaling questions” has been carried out by the authors of this paper, who have at least 5 years of expertise in the radiomic domain and are members of tasks forces for radiomic standardization such as the IBSI [10]. Each of the authors defined a list of signaling questions, which were fundamental to reproduce a study. After a round of discussion, the final list of signaling questions was obtained. If a step was not reported and could therefore not be replicated, this step was noted, and we attempted to continue to the next step with a “best guess”. This procedure was followed until all steps of the radiomics workflow was completed or guessed.

The risk probability was estimated as the number of “no’s divided by the number of “signaling” questions, expressed in percentages.

$$\mathrm{risk}\;of\;bias=\frac{\left|questions\;answered\;with\;no\right|}{\left|questions\right|}∙100\%$$

E.g. if two out of four questions were answered with a “no”, the risk of bias is calculated as:

$$\mathrm{risk}\;of\;bias=\frac{2}{4}∙100\%=50\%$$

Supplementary tables 2a-2b show the risk assessment sheets for human and phantom studies, respectively. The only differences between human and phantom studies can be found in section A. This radiomic checklist was proposed in order to tackle the limitations of using only “quality scores” to evaluate such a complex question as methodological quality.

### 2.3 Outcomes and prioritization

The primary outcome of this review was the degree of repeatability/reproducibility of a radiomic feature. The secondary outcomes were the impact of image acquisition and reconstruction settings, preprocessing steps, and tumor segmentation on the reliability/reproducibility of radiomic features. Additional outcomes were the metrics used for reporting on reliability/reproducibility. Finally, the radiomic reporting checklist was used to evaluate the quality of reporting of analyzed studies.

### 2.4 Risk of bias in individual studies

Two reviewers independently reviewed the studies. In a discussion round, both observers merged their results. This step was performed in order to avoid that the results were biased based on single reviewer’s judgement. Forced consensus was used.

## 3 Results

### 3.1 Literature search

**Fig. 1:**
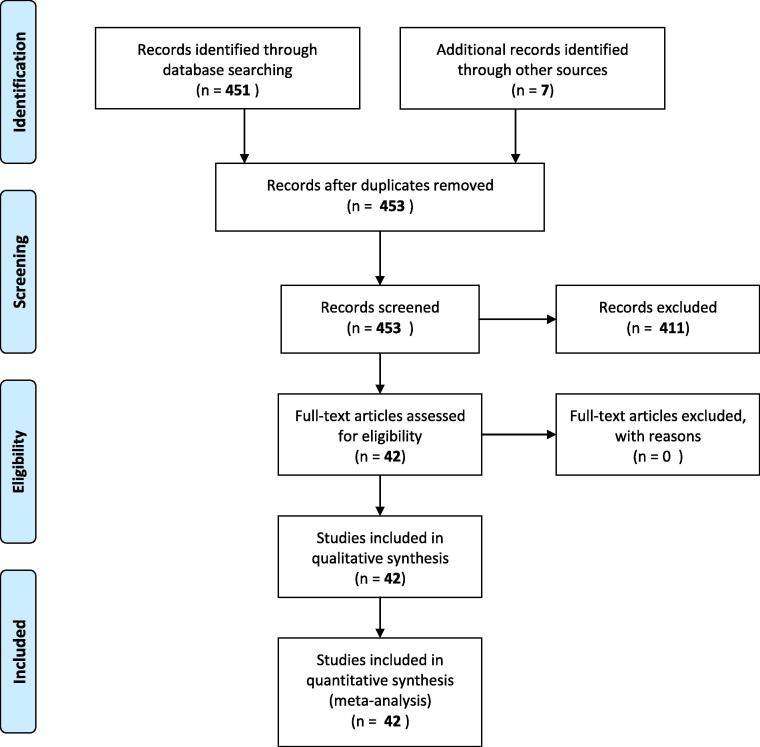
Preferred Reporting Items for Systematic Reviews and Meta-Analyses (PRISMA) flow chart. The primary PubMed search returned 168 studies. 7 studies were added from primary knowledge. After abstract screening and full-text analysis, a total of 26 studies were included in the qualitative synthesis.

A total of 451 abstracts not retrieved from the previous review were found while searching PubMed with the aforementioned search filter. After reviewing the abstracts, 42 studies fulfilled the inclusion criteria and seven additional studies had been included as prior knowledge in the field. The PRISMA flowchart illustrating the selection process is shown as Fig. 1.

Of these 42 studies, 29 studies were clinical (human subjects) studies. A summary on the study characteristics are displayed in Table 3a. Eight of these studies reported on PET, nine on MR, and twelve on CT images, with only one prospective study [11]. The number of patients included in the studies ranged from a minimum of 14 to a maximum of 465. Five consisted of multi-institutional studies. Most of the studies were focused on reproducibility, with one investigating both reproducibility and repeatability [12].

The other thirteen studies used imaging phantoms to assess repeatability/reproducibility. The general characteristics are summarized in Supplementary table 3b. Two studies reported on MR, six on PET and five on CT images. All studies were retrospective, with one study that provided a publicly available dataset [13]. Six phantom articles reported on feature reproducibility and seven on repeatability.

Five clinical studies [14], [15], [16], [17] and two phantom studies [13] made their image dataset publicly available or used a publicly available dataset. A second phantom study used a digital phantom that was already publicly available [18].

17 patient and five phantom studies used publicly available software to calculate radiomic feature values. Pyradiomics was used in eight human studies and one phantom study, and was the most frequently used software [19]. Three patient studies used the open-source software CGITA [20], two patient studies used MaZda [21], one phantom study used LifeX [22], and one phantom and one patient study used IBEX [23]. Furthermore, three studies used open-source code written in Matlab. Six human and three phantom studies failed to mention the programming language used [24].

The most frequently used metric to assess reproducibility was the ICC which was used in 19 human studies and four phantom studies. Five human studies and one phantom study used the Concordance Correlation Coefficient (CCC), however a total of four human studies and two phantom studies used other metrics not described above. Some studies used more than one metric to assess feature stability. In general, the cut-off value to dichotomize stable versus unstable features were highly heterogeneous across studies. One study failed to mention the cut-off value for the ICC, while the threshold for “excellent feature” stability differed from 0.5 to 0.85 in the other studies. One study used the half-width of the ICC confidence interval as threshold for repeatable features. CCC values above 0.8, above 0.7 or equal or higher than 0.85 were considered as robust.

### 3.2 Factors impacting radiomic feature values

The variety of analyzed settings makes it difficult to draw a general conclusion. In summary, all patient studies confirm that differences in image acquisition, reconstruction, preprocessing, and discretization have an impact on radiomic feature values [11], [15], [24], [25], [26], [27], [28], [29], [17], [30]. While the sensitivity to these factors is feature dependent, this sensitivity is found for all cancer types and imaging modalities. To mitigate this effect, Park et al. demonstrated that CT feature reproducibility can be improved by using a CNN-based super resolution algorithm [31]. For CT studies, Erdal et al. pointed out that a slice thickness of 2 mm leads to the most accurate shape features [32]. It remains unclear if radiomic features extracted from images acquired under different conditions also lead to different conclusions. In one head and neck PET study, strong dependencies of radiomic features with respect to digital image pre-processing parameters was shown, but these differences were not important enough to affect the prognostic power of radiomic features. Same conclusion holds in the study about nasopharyngeal carcinoma [25].

Many studies also reported on the sensitivity of radiomic features to differences in segmentation [12], [14], [16], [33], [34], [35], [36], [37], [38], [39], [40], [41], [42], [43], [55]. Also here, the robustness is feature dependent. Some studies demonstrated that the majority of features **was** stable to differences in tumor segmentation for lung [39] and oesophageal cancer. However, in one lung PET study [38], the shape metric sphericity was found to change its prognostic value when using different delineations. **Also** in CT studies, contours delineated by different clinicians impacted the prognostic value of radiomic features. For head and neck cancer, in one PET and one MR study features were found to be very sensitive to differences in tumor delineation [32], [33], [37], [44]. Overall, semi-automated and automated algorithms produced more stable results.

Individual studies showed that the interpolation method, image discretization, voxel size and motion blurring had an effect on radiomic feature values [32], [45], [46], [47], [48] as listed in detail in the Supplementary material.

Also results from PET phantom studies agreed that image reconstruction protocols (in particular matrix size) had strong impact on feature reproducibility, but with different level of sensitivity per feature categories. In [49], the authors pointed out that smaller volumes seem to result in lower repeatability of feature values. In contrast to these results, Ger et al. reported that most features resulted in a good or excellent reliability when the phantom was scanned on the same scanner with different acquisition protocols [50].

All CT phantom studies focused on differences in acquisition settings such as tube current and agreed that difference in acquisition protocols strongly impact feature reproducibility. However, it is difficult to draw a consensus on which features are stable: The authors in [51] demonstrated that the use of delta radiomic features, i.e. differences of feature values between two different scans of the same patient, increases the repeatability of features.

In one MR phantom study, Baessler et al. [52] investigated both reproducibility and repeatability of radiomic features using a physical phantom scanned using different sequences. The investigators showed that radiomic features extracted from FLAIR (Fluid Attenuated Inversion Recovery) images were more repeatable than features from T1- and T2-weighted images.

### 3.3 Stable feature categories

As stated above, the variety of tumor types and investigated parameters makes it difficult to identify features which are in general robust. The feature groups found to be stable by the majority of studies were statistical and morphological features, as well as GLCM and GLRLM features, while GLSZM and NGLDM features were found to be less robust. However, a few studies reported the contrary: Yang et al. found in simulated PET lung cancer data that NGLDM features were the most robust, while GLCM features were the least robust feature group [53]. One CT phantom study showed that statistical features are less robust than textural features [54]. Baessler et al. also showed in their MR phantom study that GLSZM features were more robust than GLCM features [52]. One study showed that features extracted from Fourier transformed images are the most robust, a feature group none of the other papers investigated. The authors in [51] demonstrated that the use of delta radiomic features, i.e. differences of feature values between two different scans of the same patient, increases the repeatability of features.

### 3.4 Radiomic reporting checklist

Supplementary tables 4a-5b summarizes the radiomic reporting checklist risk assessment for clinical and phantom studies. None of the studies scored a zero-risk bias probability. An example of the check-list can be found in the Supplementary material.

For clinical studies, the lowest median risk was achieved in the “study design” domain. Information about the creation of the binary mask (question B4-imaging domain) was the least reported item with only one study providing detailed information. Twelve patient studies missed to report on feature specific parameters (questions C5-C6-radiomic pipeline domain) such as feature aggregation and. In contrast, only three patient studies missed to provide any detail of the software which is a clear improvement when compared with the previous review.

For phantom studies, there were no risks of biases in the “study design” and “statistical analysis” domains. Compared to clinical studies a) all but one phantom study included a table reporting all statistical results (signaling question E3- data and metadata availability domain), and b) all but two studies failed to report on how the binary mask was created from the segmentation (question B4-imaging domain).

## 4 Discussion

The studies included in this review confirm almost all findings of the previous published review. Major issues remain the little number of studies that made their data publicly available, the heterogeneity of used metrics and cut-off values for the assessment of feature robustness, and the lack of detailed reporting. To ease the way to reproduce a study, we invite again the radiomics community to make their data and metadata publicly available. The variability in metrics and cut-offs used to categorize the features into good/poor reproducibility/repeatability, makes it difficult to compare the results of the studies. While there is no evidence that a specific metric should be used for analysis, a description about the metrics, as well as statistical hypothesis underlying the data analysis and specific cut-offs applied should be reported to guarantee the transparency and the reproducibility of the study. We also strongly recommend the users to append as supplementary material the raw results of the analysis to facilitate meta-analyses. We strongly advise to follow guidelines provided by the TRIPOD (transparent reporting of a multivariable prediction model for individual prognosis or diagnosis) statement for a transparent reporting of model design, development and evaluation.

Even though it was hard to get a consensus on which features are repeatable/reproducible, all studies agreed that reconstruction settings, image noise, and segmentation method have a high impact on radiomic feature values for all imaging modalities. This implies that multi-center radiomic studies require harmonized images in terms of image reconstruction setting and signal to noise ratio. Using images from different centers without harmonizing the images itself can lead to wrong conclusions. Further harmonization can be achieved with correction methods applied before feature extraction such as e.g. resampling the images to cubic voxels or by standardizing images via post-processing such as by histogram equalization of MR images or post-reconstruction smoothing [56], [57]. Moreover, radiomics harmonization can be achieved by image domain adaptation to reduce the influence of image acquisition settings as Chen et al showed in a simulation study [58]. However, it still has to be validated if these methods can be used for radiomic analysis as it might be that by standardizing the images important textural information gets lost.

Additionally to the standardization of images, there are methods aligning radiomic features. One of the most popular methods is the so-called ComBat, which has been applied to CT [59] and PET features [57], but still requires a large-scale validation. However, even though, these algorithms to correct for multi-center effects are being developed, it is still important to keep this correction as small as possible. Therefore, when using multi-centric data, it is essential that the images are as comparable as possible in terms of image acquisition. Moreover, patient cohorts across institutions/scanners should be comparable, i.e. the number of patients with a positive/negative outcome should be comparable across the datasets of each institution/scanner type. Otherwise the findings using a radiomic model can be due to inter-scanner differences of radiomic features and not to differences caused by variability in tumor characteristics.

To ensure a valid and reproducible analysis of PET studies, it should be carefully checked if reported tracer dose and uptake time are correct and the conversion from image data in Bq/ml to SUV units is accurate. If the liver is displayed in the image, this can be checked by drawing a 3 cm^2^ in the liver and verifying that the mean SUV inside the ‘liver’ sphere is in the range between 1.5 and 2.5. Higher/lower values are an indication for calibration or other errors and these images should be verified, corrected or excluded from the analysis. If the liver is not displayed in the image such as for brain images, a digital reference object can be used to verify the correct conversion to SUV as proposed by Pierce et al. [60]

To minimize the effect of different segmentations, a (semi-) automatic segmentation method might be preferred, as automatic approaches reduce inter-observer variability and yield a higher reproducibility than manual segmentations [61]. The most suitable segmentation method for radiomic analysis has to be identified what has to be done for each imaging modality and cancer type separately. Likely, several segmentation methods will be a good candidate as they yield similar accuracy and repeatability.

Regarding the quality of reporting, we invite the users to provide not only their software but also the metadata associated with it such as information about the programming language. In general, we recommend that each software used for feature calculation should be tested if it complies with the benchmarks provided by IBSI. In this way, feature values extracted by different software packages become comparable which is one important step in the standardization of radiomic feature values.

However, also many studies included in this review missed to report details of preprocessing steps. This missing information has the consequence that the study itself becomes non reproducible and the results are not comparable with other studies. In conclusion, to make radiomic studies comparable across centers, pre-processing steps should be standardized for each imaging modality as suggested by Park et al. [31]. This includes the discretization method as well as the bin number/bin width of choice as it has an impact on radiomic feature values [62], [63]. Since radiomic features can be sensitive to differences in voxel size, it is recommended to interpolate the images before feature extraction to an isotropic voxel size. This step and the used interpolation method should be reported if applied and the radiomic community should agree on which kind of resampling is the preferred one. Almost all studies did not report any information related to the generation of the binary mask from the original data. However, different software tools are available to go from a contour to the final binary mask, therefore it is important to state it.

Most studies reported on the robustness of first order and local textural features such as GLCM and GLRLM features, while global textural features (such as GLSZM features) were found to be less robust.

One limitation of the current review is that it was not possible to draw a general conclusion on which features are reproducible and can be used in the clinic what was one of the original aims of this review. The variety of analyzed settings and used metrics makes it impossible to perform a quantitative synthesis of the analyzed articles. Unfortunately, since the last review, the radiomics community did not came to a consensus on which metric is the most adequate to use in a radiomics setting.

## Funding

This work is part of the research program STRaTeGy with project number 14929, which is (partly) financed by the Netherlands Organization for Scientific Research (NWO).

## Declaration of Competing Interest

Andre Dekker reports grants from Varian Medical Systems, personal fees from Medical Data Works BV, personal fees from UHN Toronto, personal fees from Hanarth Fund, personal fees from Johnson & Johnson, outside the submitted work; In addition, Andre Dekker has a patent Systems, methods and devices for analyzing quantitative information obtained from radiological images US Patent 9721340 B2 issued.
