# Supplementary material for: A systematic review and quality of reporting checklist for repeatability and reproducibility of radiomic features
Source: Phys Imaging Radiat Oncol. 2021 Nov 9;20:69–75. doi: 10.1016/j.phro.2021.10.007 (PMC8591412; doi:10.1016/j.phro.2021.10.007)
Supplement: Supplementary data 1 [file mmc1.docx]

**Supplemental Material**

**Tables**

**Table 1a**: overview of the radiomic reporting checklists for phantom and clinical studies. The checklist comprises 22 items of 4 main forms for clinical and phantom studies, respectively.

| **Form A (clinical studies)– Study and Population info** | | **Form B (clinical studies) and Form F (phantom studies)– Image Acquisition and ROI(s) info** | |
| --- | --- | --- | --- |
| **A.1**  **# of Subjects** | - Clearly state the number of subjects and patient demographics (e.g. disease type, stage, purpose of scan (baseline scan/response scan) , PET tracer) that were included in the study and used for the analysis. | **B.1**  **Modality** | - Clearly state the imaging modality used to image patients. In case of MR, the sequence(s) used for the analysis should be mentioned |
| **A.2**  **Data**  **Collection** | - Clearly state if the data were collected within a single institution on a single scanner/various scanners or within multiple institutions. Provide included scanner types and name of the institutions involved in the study - Clearly state the inclusion / exclusions criteria used to select the cohort. - Clearly state the type of data that has been collected: images, clinical data or other sources (e.g. -omics) - Describe how data quality checks were performed and if data not fulfilling describe requirements were dropped | **B.2**  **Acquisition Settings** | - Append to the study a table providing major details on the acquisition settings   - The minimum list of imaging metadata for CT is provided in Appendix B.2.1   - The minimum list of imaging metadata for PET is provided in Appendix B.2.2   The minimum list of imaging metadata for MR is provided in Appendix B2.3 |
| **A.3**  **Data**  **Availability** | - Clearly state if the data is: publicly available, private or available under request - If public, the authors should provide a permanent link where the data can be accessed - If available under request, the authors should provide the name of the PI who can be contacted for data inquiry | **B.3**  **Considered ROI(s)** | - Clearly state the ROI(s) from which radiomic features were extracted - In case of radiation oncology target volumes provide standard nomenclature such as GTV, CTV or PTV - In case arbitrary ROI(s) are defined, a detailed explanation of how they were obtained is required. If this was obtained by morphological operation such as erosion or dilation, we recommend to briefly describe the algorithm used |
| **A.4**  **Study**  **Design** | - Clearly state if the study design was retrospective or prospective - For both cases include the period in which data are / were collected. - If the study involves the usage of one of more follow-ups scans the time-interval should be provided | **B.4**  **Delineation**  **Details** | - Describe how the ROI(s) were defined. Categories: manual, semi-automated or automated delineations   - Manual: provide number of observers expertise and role. Clearly describe the workstation used for the delineations   - Semi-automated/ Automated: provide references or description of the algorithm used. If possible, refer to the persistent link where the algorithm can be found - Describe how the quality of delineations was verified and what is the gold standard. - In case of delineations that are co-registered from different modalities not used for the analysis we recommend providing information about the workstation or algorithm used for the co-registration and a description on how the co-registration quality was verified |
| **A.5**  **Anatomical**  **site and**  **clinical info** | - Append to the study a table providing information about the cohort. This should include:   - Anatomical site of the disease   - Histological information   - Distribution of tumour staging   - All the clinical variables used in the study and their distribution in the cohort | **B.5**  **Binary Mask details** | - Provide name of the algorithm used to create the binary mask   Define the final format in which images and binary masks were transformed prior to features’ extraction |
| **A.6**  **Cohort**  **Demographics** | - Append to the study a paper including demographical information about the cohort. Minimum information required are:   - Age distribution   - Gender distribution | **B.6**  **Pre-Processing**  **Details** | - Provide information about any-processing step applied prior to features extraction. Please refer to the following chapters of the IBSI document   - Post-acquisition processing (chapter 2.2)   - Interpolation (chapter 2.3)   - Re-segmentation (chapter 2.5) - Digital imaging filters: provide information if original images were filtered (e.g. Wavelet filters) |
| **Form C (clinical studies) and Form G (phantom studies) – Software and Feature Info** | | **Form D (clinical studies) and Form H (phantom studies) Statistical Analysis Info** | |
| **C.1/G.1**  **Software Name** | - Provide the name of the software - Specify the version of the software - Describe if the software has been benchmarked to the IBSI standards - If the documentation of the software is available we recommend to provide a permanent link | **D.1/H.1**  **Metric** | - Clearly state the statistical metric(s) used for the analysis. For metrics that required multiple definitions, such as ICC, all the details should be provided - For newly defined metrics provide definitions and formulas |
| **C.2/G/2**  **Software**  **Availability** | - Describe if the software is open source or proprietary   - In case of open source software provide the permanent link where the software can be accessed.   - In case of proprietary software provide the name of the developer | **D.2/H.2**  **Cut-off** | - Clearly describe cut-offs used to defined different reproducibility and repeatability categories |
| **C.3/G.3**  **Programming Language** | - Describe the programming language of the software - Clearly state the version of the programming language | **D3/H.3**  **Results Availability** | - The raw statistical results (e.g. ICC values) are appended to the study as supplementary material |
| **C.4/G.4**  **Feature Classes** | - Describe the feature classes used in the analysis. Adhere to the nomenclature provided in the IBSI document (chapters 3.1-3.11) - If new feature classes are defined, references to their description should be provided |  | |
| **C.5/G.5**  **Number of features** | - Clearly state the number of features computed for each category | **Form E (phantom studies) – Study and Phantom Info** | |
| **C.6/G.6**  **Feature Description** | - Describe the mathematical definitions of the features   - If previously defined features are used, the link to the formulas should be provided   - If new features are defined, the formulas should be provided as supplementary material | **E.1**  **# of Scans** | - Clearly state the number of phantom scans acquired in the analysis |
| **C.7/G.7**  **Feature Specific Parameters** | - Describe the particular settings used to compute features:   - Intensity Discretization (chapter 2.7)   - Feature aggregation (chapter 2.8) | **E.2**  **Data**  **Collection** | - Clearly state if the data were collected within a single institution on a single scanner/various scanners or within multiple institutions. Provide included scanner types and name of the institutions involved in the study - Clearly state the inclusion / exclusions criteria used to select the data. - Clearly state the type of data that has been collected   Describe how data quality checks were performed and if data not fulfilling describe requirements were dropped |
|  | | **E.3**  **Data Availability** | - Clearly state if the data is: publicly available, private or available under request - If public, the authors should provide a permanent link where the data can be accessed   If available under request, the authors should provide the name of the PI who can be contacted for data inquiry |
|  |  | **E.4**  **Study**  **Design** | - Clearly state if the study design was retrospective or prospective - For both cases include the period in which data are / were collected.   If the study involves the usage of one of more follow-ups scans the time-interval should be provided |
|  |  | **E.5**  **Phantom**  **Characteristics** | - Clearly describe the model of the phantom and the manufacturer - For in-house phantom describe the institution that built the phantom   Clearly describe the material of the phantom, the number of plugs and their material |

**Table 1b**: appendix to the check list. To further guide the authors in the reporting of major imaging acquisition parameters, we provided detailed guidelines of the DICOM metadata to be reported.

| **Appendix – Image Acquisition Details** | |
| --- | --- |
| B.2.1  CT | - Scanner manufacturer: DICOM TAG (0008,0070) - Scanner model: DICOM TAG (0008,1090) - Slice thickness: DICOM TAG (0018,0050) - Pixel spacing: DICOM TAG (0028,0030) - X-ray tube current: DICOM TAG (0018,1151) - Exposure: DICOM TAG (0018,1152) - Convolution kernel: DICOM TAG (0018,1210) |
| B.2.2  PET | - Scanner manufacturer: DICOM TAG (0008,0070) - Scanner model: DICOM TAG (0008,1090) - Slice thickness: DICOM TAG (0018,0050) - Pixel spacing: DICOM TAG (0028,0030) - Acquisition date: DICOM TAG (0008, 0022) - Acquisition time: DICOM TAG (0008,0032) - Series time (for decay correction): (0008, 0031) - Patient's sex: DICOMTAG (0010, 0040) - Patient’s weight: DICOMTAG (0010, 1030) - Patient’s size: DICOMTAG (0010, 1020) - Units: DICOMTAG (0054, 1001) - Actual frame duration: DICOMTAG (0018, 1242) - Decay Correction: DICOMTAG (0054, 1102) - Radiopharmaceutical: DICOMTAG (0018, 0031) - Radiopharmaceutical start date time: DICOMTAG (0018, 1078) - Radionuclide total dose: DICOM TAG (0018,1074) - Number of iterations: DICOM TAG (0018,9739) - Number of subsets: DICOM TAG (0018,9740) - Processing function: DICOM TAG (0018,5020) - Convolution kernel: DICOM TAG (0018,1210) - Reconstruction algorithm: DICOM TAG (0018,9756) (in case that PSF and TOF reconstructions were performed that are not mentioned in the DICOMTAG, the information about these reconstructions should be extracted by checking the scanner specific reconstruction protocol. The same applied to any other missing information regarding reconstruction settings) |
| B.2.3  MR | - Scanner manufacturer: DICOM TAG (0008,0070) - Scanner model: DICOM TAG (0008,1090) - Slice thickness: DICOM TAG (0018,0050) - Pixel spacing: DICOM TAG (0028,0030) - Scanning sequence: DICOM TAG (0018,0020) - Sequence variant: DICOM TAG (0018,0021) - Scan options: DICOM TAG (0018,0022) - Repetition time: DICOM TAG (0018,0080) - Echo time: DICOM TAG (0018,0081) - Magnetic field strength: DICOM TAG (0018.0087) |

**Table 2a.** Overview of the checklist and signalling questions for the clinical studies

| Item | Domain | Yes | No | Unclear |
| --- | --- | --- | --- | --- |
| *A1. Were the selection criteria of the clinical cohort(s) clearly described?* | Study Design | () | () | () |
| *A1. Was the number of patients enrolled in this study clearly stated?* | Study Design | () | () | () |
| *A3. Were the institution(s) involved in the study clearly mentioned in the manuscript?* | Study Design | () | () | () |
| *A4. Were clinical and population variables available in the study?* | Study Design | () | () | () |
|  | | | | |
| *B1. Were the imaging modalities used to acquire scans of the patients clearly described?* | Imaging | () | () | () |
| *B2. Were the image acquisition settings for the images used in the analysis clearly described?* | Imaging | () | () | () |
| *B3. Were the ROI(s) used to extract radiomic features clearly described and how they were segmented?* | Imaging | () | () | () |
| *B4. Was the algorithm / mathematical function used to transform contours to masks provided?* | Imaging | () | () | () |
|  |  |  |  |  |
| *C1. Were the image pre-processing steps, prior to feature extraction, clearly described?* | Radiomic  Pipeline | () | () | () |
| *C2. Was the software used for radiomic computations described (version, developers, programming language)?* | Radiomic  Pipeline | () | () | () |
| *C3. Were the radiomic feature classes and single features described together with their formulas?* | Radiomic Pipeline | () | () | () |
| *C4. Was the total number of features extracted clearly mentioned?* | Radiomic  Pipeline | () | () | () |
| *C5. Was the feature aggregation algorithm specified?* | Radiomic  Pipeline | () | () | () |
| *C6. Was the feature quantization algorithm specified?* | Radiomic  Pipeline | () | () | () |
|  |  |  |  |  |
| *D1. Were the statistical tests and metrics used to evaluate features’ reproducibility / repeatability clearly stated?* | Statistical  Analysis | () | () | () |
| *D2. Were the reference standards to define levels of reproducibility specified?* | Statistical  Analysis | () | () | () |
|  |  |  |  |  |
| *E1. Were the images and segmentation shared as public data?* | Data and metadata  Availability | () | () | () |
| *E2. Was the software shared as open-source code?* | Data and metadata  Availability | () | () | () |
| *E3. Were the results of the statistical analysis shared as public data?* | Data and metadata  Availability | () | () | () |

**Table 2b.** Overview of the checklist and signalling questions for the phantom studies

| Item | Domain | Yes | No | Unclear |
| --- | --- | --- | --- | --- |
| *A1. Were the details about the phantom used in the study clearly specified?* | Study Design | () | () | () |
| *A1.* *Was the number of acquired scans clearly stated?* | Study Design | () | () | () |
| *A3. Were the institution(s) involved in the study clearly mentioned in the manuscript?* | Study Design | () | () | () |
|  | | | | |
| *B1. Were the imaging modalities used to acquire scans of the phantom(s) clearly described?* | Imaging | () | () | () |
| *B2. Were the image acquisition settings for the images used in the analysis clearly described?* | Imaging | () | () | () |
| *B3. Were the ROI(s) used to extract radiomic features clearly described and how they were segmented?* | Imaging | () | () | () |
| *B4. Was the algorithm / mathematical function used to transform contours to masks provided?* | Imaging | () | () | () |
|  |  |  |  |  |
| *C1. Were the image pre-processing steps, prior to feature extraction, clearly described?* | Radiomic  Pipeline | () | () | () |
| *C2. Was the software used for radiomic computations described (version, developers, programming language)?* | Radiomic  Pipeline | () | () | () |
| *C3. Were the radiomic feature classes and single features described together with their formulas?* | Radiomic Pipeline | () | () | () |
| *C4. Was the total number of features extracted clearly mentioned?* | Radiomic  Pipeline | () | () | () |
| *C5. Was the feature aggregation algorithm specified?* | Radiomic  Pipeline | () | () | () |
| *C6. Was the feature quantization algorithm specified?* | Radiomic  Pipeline | () | () | () |
|  |  |  |  |  |
| *D1. Were the statistical tests and metrics used to evaluate features’ reproducibility / repeatability clearly stated?* | Statistical  Analysis | () | () | () |
| *D2. Were the reference standards to define levels of reproducibility* | Statistical  Analysis | () | () | () |
|  |  |  |  |  |
| *E1. Were the images and segmentation shared as public data?* | Data and metadata  Availability | () | () | () |
| *E2. Was the software shared as open-source code?* | Data and metadata  Availability | () | () | () |
| *E3. Were the results of the statistical analysis shared as public data?* | Data and metadata  Availability | () | () | () |

**Table 3a**. summary table of the 29 clinical studies included in the analysis. Abbreviations: NA refers to information that could not be retrieved from the manuscript; FO: First order features; SM: Shape Metric features; TA: Texture Analysis features; LOG: Logarithmic filtered features; WF: Wavelet Filtered features; SR: Square Root filtered features; SQ: Square filtered features; EXP: Exponential filtered features; GRAD: Gradient filtered features; FBN: Fixed-bin Number quantization; FBW: Fixed-Bin Width quantization; CCC: Concordance Correlation Coefficient; ICC: Intra-class Correlation Coefficient; PPV: Positive Predictive Value; TRR: Test-

| Reference | Study  Design | Data  Availability | Multi  Institutional | Cancer type  (# subjects) | Modality | Delineation  Methods | Software Name/ Programming  Language  (Open Source) | Features  (classes) | Texture features quantization method | Statistical  Metric |
| --- | --- | --- | --- | --- | --- | --- | --- | --- | --- | --- |
| (Altazi et al., 2017a) | Retrospective | No | No | Cervix  (88) | PET | Manual  Semi-auto | In-house  NA | 79  (FO,SM,TA) | FBN | MPD  ICC |
| (Fiset et al., 2019) | Retrospective | No | No | Cervix  (62) | MR | Manual | Pyradiomics/  Python3.7  (Yes) | 1761  (FO,SM,  TA,LOG,WF) | FBW | ICC |
| (Lv et al., 2018) | Retrospective | No | No | HN  (106) | PET | Semi-auto | In-house/  Matlab  (Yes) | 53  (TA) | FBW | ICC |
| (Belli et al., 2018) | Retrospective | No | No | HN / Pancreatic  (50) | PET | Semi-auto | CGITA  Matlab  (Yes) | 73  (FO,TA) | FBW | ICC |
| (Xia et al., 2018a) | Retrospective | Yes | No | Liver  (38) | CT | Semi-auto | NA | 37  (FO,SM) | NA | ICC |
| (Carles et al., 2018) | Retrospective | No | No | Lung  (31) | PET | Semi-auto | In-house  NA | 36  (FO,SM,TA) | FBN  FBW | ICC |
| (Hatt et al., 2018a) | Retrospective | No | Yes | Lung  (263) | PET | Auto | NA | 2  (SM) |  | PPV  Spearman |
| (Johnson et al., 2017) | Retrospective | No | No | Lung  (26) | PET | Semi-auto | NA | 10  (FO,SM) |  | Pearson |
| (Zhuang et al., 2019) | Prospective | No | No | Lung  (61) | PET | Semi-auto | Pyradiomics/  Python 3.7  (Yes)  In-house  NA  (No) | 10  (FO,TA) | NA | TRR |
| (Lafata et al., 2018) | Retrospective | No | No | Lung  (97) | CT | Semi-auto | In-house  NA | 43  (FO,TA) | NA | CCC |
| (Haga et al., 2018b) | Retrospective | No | No | Lung  (40) | CT | Manual  Semi-auto | In-house  Matlab  (Yes) | 476  (FO,SM,TA) | FBW | ROC-AUC |
| (Takeda et al., 2017) | Retrospective | No | No | Lung  (26) | PET | Manual | CGITA  Matlab  (Yes) | 7  (FO,TA) | FBN | ICC |
| (Schwier et al., 2018) | Retrospective | Yes | No | Prostate  (14) | MR | Semi-auto | Pyradiomics  Python 3.7  (Yes) | (FO,SM,TA,LOG,  SR,SQ,EXP,WF) | FBN | ICC |
| (Traverso et al., 2019) | Retrospective | No | Yes | Rectum  (56) | MR | Manual | Pyradiomics  Python 3.7  (Yes) | 70  (FO,SM,TA) | FBW | CCC  ICC |
| (Bektas et al., 2019) | Retrospective | No | No | Renal  (53) | CT | Manual | MaZda  C++  (No) | 279  (FO,TA,GRAD,WF) | FBN | ICC |
| (Feng et al., 2018b) | Restropective | No | No | Renal  (58) | CT | Manual | CT Kinetics  NA  (No) | 42  (FO,TA) | NA | ICC |
| (Kocak et al., 2018) | Retrospective | No | Yes | Renal  (94) | CT | Manual | MaZda  NA  (No) | 275  (FO,TA,WF,AR) | NA | ICC |
| (Zhang et al., 2018) | Retrospective | No | No | Brain  (87) | MR | Manual | IBEX  Matlab  (Yes) | 1140  (FO,SM,GRAD,TA) | NA | CCC |
| (Qu et al., 2019) | Retrospective | No | No | Esophageal  (181) | MR | Manual | In-house  Matlab  (No) | 1578  (FO,SM,TA,FD) | NA | ICC |
| (Erdal et al., 2020) | Retrospective | No | No | Kidney  (23) | CT | Semi-automatic | MeViSLab  (Yes) | 28  (FO, gradient, GLCM, GLRLM, autoregressive, wavelet) | FBN | ICC |
| (Haarburger et al., 2020) | Retrospective | Yes | Yes | helical thoracic, kidney tumours, liver    (NA) | CT | Manual, semi-automatic | PyRadiomics  (Yes) | 89  (GLCM, GLSZM, GLRLM, NGTDM) | FBW | ICC |
| (Lee et al., 2019) | Retrospective | No | No | Lung  (260) | CT | Semi-automatic | PyRadiomics and inhouse  (Yes/No) | 252  (FO, GLCM, NGTDM) | NA | ICC |
| (Loi et al., 2019) | Retrospective | No | No | Pankreas  (39) | CT | Manual | CGITA  (Yes) | 108  (FO, GLSZM, GLCM, GLDM, GLRLM) | FBN | COV |
| (Meyer et al., 2019) | Retrospective | No | No | Liver  (78) | CT | Manual | PyRadiomics  (Yes) | 106  (FO, GLCM, GLDM, GLRLM, GLSZM, NGTDM, shape) | NA | Hierarchical clustering |
| (Moradmand et al., 2019) | Retrospective | Yes | Yes | Brain  (262) | MR | Semi-automatic | PyRadiomics  (Yes) | NA  (GLCM, GLRLM, GLDZM, GLSZM, NGTDM) | NA | CCC, ICC |
| (Park et al., 2019) | Retrospective | No | No | Lung  (106) | CT | Semi-automatic | NA | 702  (NA) | NA | CCC |
| (Tixier et al., 2019) | Retrospective | No | No | Brain  (90) | MR | Semi-automatic | CERR  (yes) | 180  (FO, shape, GLCM, GLSZM, edge maps) | FBN | ICC |
| (Yamashita et al., 2019) | Retrospective | No | No | Pankreas  (39) | CT | Manual | NA  (NA) | 266  (FO, fractal dimensions, GLCM, GLRLM, shape, angular co-occurrence , local binary patterns ) | NA | CCC, ICC |
| (Yang et al., 2020) | Retrospective | No | No | Lung  (26) | PET | Manual | NA  (NA) | 25  (GLCOM, NGLDM, GLSZM) | FBN | ICC |
| (Zhang et al., 2020) | Retrospective | No | No | Head and Neck  (384) | MR | Manual | Matlab code  (Yes) | 2068  (FO, GLCM, GLRLM, GLSZM, NGTDM) | NA | ICC |

**Table 3b**: summary table of the 13 phantom studies. Abbreviations: NA no information could be retrieved from the manuscript; FO: First order; SM: Shape Metric; TA: Texture Analysis; LBP: Local Binary Pattern; FD: Fractal Dimension; ACM: Angle Co-Occurrence Matrix; LI: Local Intensity; TS: Texture Spectrum; FBN: Fixed-Bin Number quantization; FBW: Fixed-Bin Width quantization; CCC: Concordance Correlation Coefficient; ICC: Intra-class Correlation Coefficient; DR: Dynamic Range; COV: Coefficient Of Variation; PAD: Percentage Absolute Difference.

| Reference | Study  Design | Data  Availability | Multi  Institutional | Phantom  Model  (Manufacturer) | Modality | Delineation  Methods | Software  Name/ Programming  Language  (Open Source) | Features  (classes) | Texture features quantization method | Statistical  Metric |
| --- | --- | --- | --- | --- | --- | --- | --- | --- | --- | --- |
| (Baeßler et al., 2018) | Retrospective | No | No | NA  (In-house) | MR | Semi-auto | LIFEx/  Java  (Yes) | 45  (FO,TA) | FBN | CCC  ICC  DR |
| (Ger et al., 2019) | Retrospective | No | Yes | Hoffmann Brain Phantom | PET | Manual | IBEX  (Yes) | 23  (GLCM, GLRLM, NGTDM) | FBN, FBW | ICC |
| (Johnson et al., 2017) | Retrospective | No | No | Simulated Lung  (26) | PET | Semi-auto | NA | 10  (FO,SM) |  | Pearson |
| (Midya et al., 2018a) | Retrospective | No | No | ATOM  (CIRS) | CT | Manual | In-house/  Matlab  (No) | 248  (FO,TA,  LBP,FD, ACM) | NA | CCC |
| (Papp et al., 2019) | Retrospective | No | Yes | NEMA | PET | Semi-auto | In-house/  NA  (No) | 37  (SM,TA) | FBW | COV |
| (Pfaehler et al., 2019) | Retrospective | No | No | NEMA | PET | Manual  Semi-auto | In-house/  Matlab  (No) | 246  (FO,LI,SM, TA) | FBN  FBW | ICC |
| (Shiri et al., 2017a) | Retrospective | No | Yes | NEMA  (In-house) | PET | Semi-auto | In-house/  Matlab  (No) | 100  (FO,SM,  TA,TS) | NA | COV |
| (Varghese et al., 2019a) | Retrospective | No | Yes | CTTA  (In-house) | CT | Manual | NA | NA  (FO,TA,FFT) | FBN | PAD |
| (Yang et al., 2018b) | Retrospective | No | Yes | Digital  (In-house) | MR | Manual | NA | 23  (TA) | FBN | COV |
| (Yang et al., 2020) | Retrospective | No | No | Digital  (In-house) | PET | Manual | NA | 25  (GLCOM, NGLDM, GLSZM) | FBN | ICC |
| (Zhovannik et al., 2019a) | Retrospective | Yes | No | Gammex  (Sun Nuclear) | CT | Manual | Pyradiomics/  Python3.7  (Yes) | 92  (FO,TA) | FBW | ICC |

**Table 4a:** summary of the radiomic reporting checklist for clinical studies

| **Clinical studies (N= 29)**  **Radiomic Reporting checklist overall summary** | | | | | |
| --- | --- | --- | --- | --- | --- |
| Reference | FORM A  Study and  Population  info | FORM B  Image  Acquisition  And  ROI(s) info | FORM C  Software  And  Feature info | FORM D  Statistical  Analysis  Info | **Total**  **Reported**  **Items** |
| (Altazi et al., 2017a) | **5/6** | **4/6** | **6/7** | **2/3** | **17/22** |
| (Belli et al., 2018) | **5/6** | **4/6** | **6/7** | **2/3** | **17/22** |
| (Carles et al., 2018) | **4/6** | **4/6** | **6/7** | **3/3** | **17/22** |
| (Erdal et al., 2019) | **4/6** | **5/6** | **5/7** | **2/3** | **16/22** |
| (Feng et al., 2019) | **6/6** | **5/6** | **3/7** | **2/3** | **14/22** |
| (Fiset et al., 2019) | **6/6** | **5/6** | **6/7** | **3/3** | **21/22** |
| (Haarburger et al., 2020) | **5/6** | **4/6** | **6/7** | **2/3** | **17/22** |
| (Haga et al., 2019) | **5/6** | **5/6** | **6/7** | **1/3** | **17/22** |
| (Hatt et al., 2018a) | **5/6** | **3/6** | **4/7** | **2/3** | **14/22** |
| (Lafata et al., 2018) | **6/6** | **4/6** | **6/7** | **3/3** | **19/22** |
| (Lee et al., 2019) | **5/6** | **5/6** | **5/7** | **3/3** | **18/22** |
| (Loi et al., 2019) | **5/6** | **5/6** | **6/7** | **2/3** | **18/22** |
| (Lv et al., 2018) | **6/6** | **5/6** | **7/7** | **1/3** | **19/22** |
| (Meyer et al., 2019) | **6/6** | **5/6** | **6/7** | **2/3** | **19/22** |
| (Moradman et al., 2019) | **6/6** | **5/6** | **6/7** | **2/3** | **19/22** |
| (Saha et al., 2018) | **4/6** | **5/6** | **6/7** | **3/3** | **16/22** |
| (Schwier et al., 2018) | **6/6** | **5/6** | **7/7** | **2/3** | **20/22** |
| (Traverso et al., 2019) | **6/6** | **6/6** | **7/7** | **2/3** | **21/22** |
| (Xia et al., 2018a) | **6/6** | **5/6** | **1/7** | **2/3** | **14/22** |
| (Zhuang et al., 2019) | **6/6** | **5/6** | **2/7** | **2/3** | **15/22** |
| (Bektas et al., 2019) | **6/6** | **3/6** | **6/7** | **3/3** | **18/22** |
| (Takeda et al., 2017) | **6/6** | **4/6** | **6/7** | **3/3** | **19/22** |
| (Zhang et al., 2018) | **5/6** | **5/6** | **6/7** | **2/3** | **18/22** |
| (Kocak et al., 2018) | **6/6** | **5/6** | **5/7** | **2/3** | **18/22** |
| (Qu et al., 2019) | **6/6** | **4/6** | **5/7** | **2/3** | **17/22** |
| (Yang et al., 2018) | **6/6** | **5/6** | **4/7** | **2/3** | **17/22** |
| (Whybra et al., 2019) | **5/6** | **5/6** | **5/7** | **2/3** | **17/22** |
| (Park et al., 2019) | **6/6** | **5/6** | **3/7** | **2/3** | **16/22** |
| (Tixier et al., 2019) | **6/6** | **5/6** | **6/7** | **2/3** | **19/22** |
| (Yamashita et al., 2019) | **5/6** | **5/6** | **4/7** | **2/3** | **17/22** |

**Table 4b:** Detailed information of the reported and missing items of the radiomic reporting checklist for clinical studies: Form A reports on the domain study design; Form B on imaging, Form C on the description of the radiomic pipeline, Form D on the information about statistical analysis, and Form E on the provided information of data and metadata availability

| **Form A – Study and Population info** | | | | | | | | |
| --- | --- | --- | --- | --- | --- | --- | --- | --- |
| Reference | A.1  # of Subjects | A.2  Data  Collection | A.3  Data  Availability | A.4  Study  Design | A.5  Anatomical  site and  clinical info | A.6  Cohort  Demographics |  | **Reported**  **Items** |
| (Altazi et al., 2017a) |  |  |  |  |  |  |  | **5/6** |
| (Belli et al., 2018) |  |  |  |  |  |  |  | **5/6** |
| (Carles et al., 2018) |  |  |  |  |  |  |  | **4/6** |
| (Erdal et al., 2019) |  |  |  |  |  |  |  | **4/6** |
| (Feng et al., 2019) |  |  |  |  |  |  |  | **6/6** |
| (Fiset et al., 2019) |  |  |  |  |  |  |  | **6/6** |
| (Haarburger et al., 2019) |  |  |  |  |  |  |  | **5/6** |
| (Hatt et al., 2018a) |  |  |  |  |  |  |  | **5/6** |
| (Lafata et al., 2018) |  |  |  |  |  |  |  | **6/6** |
| (Lee et al., 2019) |  |  |  |  |  |  |  | **5/6** |
| (Loi et al., 2019) |  |  |  |  |  |  |  | **5/6** |
| (Lv et al., 2018) |  |  |  |  |  |  |  | **6/6** |
| (Meyer et al., 2019) |  |  |  |  |  |  |  | **6/6** |
| (Moradman et al., 2019) |  |  |  |  |  |  |  | **6/6** |
| (Saha et al., 2019) |  |  |  |  |  |  |  | **4/6** |
| (Schwier et al., 2018) |  |  |  |  |  |  |  | **6/6** |
| (Traverso et al., 2019) |  |  |  |  |  |  |  | **6/6** |
| (Xia et al., 2018a) |  |  |  |  |  |  |  | **6/6** |
| (Zhuang et al., 2019) |  |  |  |  |  |  |  | **6/6** |
| (Bektas et al., 2019) |  |  |  |  |  |  |  | **6/6** |
| (Haga et al., 2018b) |  |  |  |  |  |  |  | **5/6** |
| (Takeda et al., 2017) |  |  |  |  |  |  |  | **6/6** |
| (Zhang et al., 2018) |  |  |  |  |  |  |  | **5/6** |
| (Kocak et al., 2018) |  |  |  |  |  |  |  | **6/6** |
| (Qu et al., 2019) |  |  |  |  |  |  |  | **6/6** |
| (Yang et al., 2018) |  |  |  |  |  |  |  | **6/6** |
| (Whybra et al., 2019) |  |  |  |  |  |  |  | **5/6** |
| (Park et al., 2019) |  |  |  |  |  |  |  | **6/6** |
| (Tixier et al., 2019) |  |  |  |  |  |  |  | **6/6** |
| (Yamashita et al., 2019) |  |  |  |  |  |  |  | **5/6** |
|  | | | | | | | | |
| **Form B – Image acquisition and ROI(s) info** | | | | | | | | |
| Reference | B.1  Modality | B.2  Acquisition  Settings | B.3  Considered  ROI(s) | B.4  Delineation  Details | B.5  Binary Mask  Details | B.6  Pre-processing  Details |  | **Reported**  **Items** |
| (Altazi et al., 2017a) |  |  |  |  |  |  |  | **4/6** |
| (Belli et al., 2018) |  |  |  |  |  |  |  | **4/6** |
| (Carles et al., 2018) |  |  |  |  |  |  |  | **4/6** |
| (Erdal et al., 2019) |  |  |  |  |  |  |  | **5/6** |
| (Feng et al., 2019) |  |  |  |  |  |  |  | **4/6** |
| (Fiset et al., 2019) |  |  |  |  |  |  |  | **5/6** |
| (Haarburger et al., 2019) |  |  |  |  |  |  |  | **4/6** |
| (Haga et al., 2019) |  |  |  |  |  |  |  | **5/6** |
| (Hatt et al., 2018a) |  |  |  |  |  |  |  | **3/6** |
| (Lafata et al., 2018) |  |  |  |  |  |  |  | **4/6** |
| (Lee et al., 2019) |  |  |  |  |  |  |  | **5/6** |
| (Loi et al., 2019) |  |  |  |  |  |  |  | **5/6** |
| (Lv et al., 2018) |  |  |  |  |  |  |  | **5/6** |
| (Meyer et al., 2019) |  |  |  |  |  |  |  | **5/6** |
| (Moradman et al., 2019) |  |  |  |  |  |  |  | **5/6** |
| (Saha et al., 2018) |  |  |  |  |  |  |  | **5/6** |
| (Schwier et al., 2018) |  |  |  |  |  |  |  | **5/6** |
| (Traverso et al., 2019) |  |  |  |  |  |  |  | **6/6** |
| (Xia et al., 2018a) |  |  |  |  |  |  |  | **5/6** |
| (Zhuang et al., 2019) |  |  |  |  |  |  |  | **5/6** |
| (Bektas et al., 2019) |  |  |  |  |  |  |  | **3/6** |
| (Haga et al., 2018b) |  |  |  |  |  |  |  | **5/6** |
| (Takeda et al., 2017) |  |  |  |  |  |  |  | **4/6** |
| (Zhang et al., 2018) |  |  |  |  |  |  |  | **5/6** |
| (Kocak et al., 2018) |  |  |  |  |  |  |  | **5/6** |
| (Qu et al., 2019) |  |  |  |  |  |  |  | **4/6** |
| (Yang et al., 2018) |  |  |  |  |  |  |  | **5/6** |
| (Whybra et al., 2019) |  |  |  |  |  |  |  | **5/6** |
| (Park et al., 2019) |  |  |  |  |  |  |  | **5/6** |
| (Tixier et al., 2019) |  |  |  |  |  |  |  | **5/6** |
| (Yamashita et al., 2019) |  |  |  |  |  |  |  | **5/6** |
|  | | | | | | | | |
| **Form C – Software and Feature info** | | | | | | | | |
| Reference | C.1  Software  Name | C.2  Software  Availability | C.3  Programming  Language | C.4  Feature  Classes | C.5  Number of  Features | C.6  Feature  Description | C.7  Feature  Specific  Parameters | **Reported**  **Items** |
| (Altazi et al., 2017a) |  |  |  |  |  |  |  | **6/7** |
| (Belli et al., 2018) |  |  |  |  |  |  |  | **6/7** |
| (Carles et al., 2018) |  |  |  |  |  |  |  | **6/7** |
| (Erdal et al., 2019) |  |  |  |  |  |  |  | **5/7** |
| (Feng et al., 2019) |  |  |  |  |  |  |  | **5/7** |
| (Fiset et al., 2019) |  |  |  |  |  |  |  | **6/7** |
| (Haarburger et al., 2019) |  |  |  |  |  |  |  | **6/7** |
| (Hatt et al., 2018a) |  |  |  |  |  |  |  | **4/7** |
| (Lafata et al., 2018) |  |  |  |  |  |  |  | **6/7** |
| (Lee et al., 2019) |  |  |  |  |  |  |  | **5/7** |
| (Loi et al., 2018) |  |  |  |  |  |  |  | **6/7** |
| (Lv et al., 2018) |  |  |  |  |  |  |  | **7/7** |
| (Meyer et al., 2019) |  |  |  |  |  |  |  | **6/7** |
| (Moradman et al., 2019) |  |  |  |  |  |  |  | **6/7** |
| (Saha et al., 2018) |  |  |  |  |  |  |  | **6/7** |
| (Schwier et al., 2018) |  |  |  |  |  |  |  | **7/7** |
| (Traverso et al., 2019) |  |  |  |  |  |  |  | **7/7** |
| (Xia et al., 2018a) |  |  |  |  |  |  |  | **1/7** |
| (Zhuang et al., 2019) |  |  |  |  |  |  |  | **2/7** |
| (Bektas et al., 2019) |  |  |  |  |  |  |  | **6/7** |
| (Haga et al., 2018b) |  |  |  |  |  |  |  | **6/7** |
| (Takeda et al., 2017) |  |  |  |  |  |  |  | **6/7** |
| (Zhang et al., 2018) |  |  |  |  |  |  |  | **6/7** |
| (Kocak et al., 2018) |  |  |  |  |  |  |  | **5/7** |
| (Qu et al., 2019) |  |  |  |  |  |  |  | **5/7** |
| (Yang et al., 2018) |  |  |  |  |  |  |  | **4/7** |
| (Whybra et al., 2019) |  |  |  |  |  |  |  | **5/7** |
| (Park et al., 2019) |  |  |  |  |  |  |  | **3/7** |
| (Tixier et al., 2019) |  |  |  |  |  |  |  | **6/7** |
| (Yamashita et al., 2019) |  |  |  |  |  |  |  | **4/7** |
|  | | | | | | | | |
| **Form D – Statistical Analysis Info** | | | | | | | | |
| Reference | D.1  Metric | D.2  Cut-off(s) | D.3  Results  Availability |  |  |  |  | **Reported**  **Items** |
| (Altazi et al., 2017a) |  |  |  |  |  |  |  | **2/3** |
| (Belli et al., 2018) |  |  |  |  |  |  |  | **2/3** |
| (Carles et al., 2018) |  |  |  |  |  |  |  | **3/3** |
| (Erdal et al., 2019) |  |  |  |  |  |  |  | **2/3** |
| (Feng et al., 2019) |  |  |  |  |  |  |  | **2/3** |
| (Fiset et al., 2019) |  |  |  |  |  |  |  | **3/3** |
| (Haarburger et al., 2019) |  |  |  |  |  |  |  | **2/3** |
| (Hatt et al., 2018a) |  |  |  |  |  |  |  | **2/3** |
| (Lafata et al., 2018) |  |  |  |  |  |  |  | **3/3** |
| (Lee et al., 2019) |  |  |  |  |  |  |  | **3/3** |
| (Loi et al., 2019) |  |  |  |  |  |  |  | **2/3** |
| (Lv et al., 2018) |  |  |  |  |  |  |  | **1/3** |
| (Meyer et al., 2019) |  |  |  |  |  |  |  | **2/3** |
| (Moradman et al., 2019) |  |  |  |  |  |  |  | **2/3** |
| (Saha et al., 2018) |  |  |  |  |  |  |  | **3/3** |
| (Schwier et al., 2018) |  |  |  |  |  |  |  | **2/3** |
| (Traverso et al., 2019) |  |  |  |  |  |  |  | **2/3** |
| (Xia et al., 2018a) |  |  |  |  |  |  |  | **2/3** |
| (Zhuang et al., 2019) |  |  |  |  |  |  |  | **2/3** |
| (Bektas et al., 2019) |  |  |  |  |  |  |  | **3/3** |
| (Haga et al., 2018b) |  |  |  |  |  |  |  | **0/3** |
| (Takeda et al., 2017) |  |  |  |  |  |  |  | **3/3** |
| (Zhang et al., 2018) |  |  |  |  |  |  |  | **2/3** |
| (Kocak et al., 2018) |  |  |  |  |  |  |  | **2/3** |
| (Qu et al., 2019) |  |  |  |  |  |  |  | **2/3** |
| (Yang et al., 2018) |  |  |  |  |  |  |  | **2/3** |
| (Whybra et al., 2019) |  |  |  |  |  |  |  | **2/3** |
| (Park et al., 2019) |  |  |  |  |  |  |  | **2/3** |
| (Tixier et al., 2019) |  |  |  |  |  |  |  | **2/3** |
| (Yamashita et al., 2019) |  |  |  |  |  |  |  | **2/3** |

**Table 5a:** summary of the radiomic reporting checklist for phantom studies

| **Phantom studies (N= 8)**  **Radiomic Reporting checklist overall summary** | | | | | |
| --- | --- | --- | --- | --- | --- |
| Reference | FORM E  Study and  Population  info | FORM F  Image  Acquisition  And  ROI(s) info | FORM G  Software  And  Feature info | FORM H  Statistical  Analysis  Info | **Total**  **Reported**  **Items** |
| (Baeßler et al., 2018) | **6/6** | **6/6** | **6/7** | **3/3** | **21/22** |
| (Ger et al., 2017) | **4/6** | **5/6** | **6/7** | **2/3** | **17/22** |
| (Johnson et al., 2017) | **6/6** | **5/6** | **3/7** | **1/3** | **14/22** |
| (Midya et al., 2018) | **6/6** | **6/6** | **6/7** | **3/3** | **21/22** |
| (Nardone et al., 2018) | **6/6** | **5/6** | **6/7** | **3/3** | **20/22** |
| (Papp et al., 2019) | **6/6** | **5/6** | **4/7** | **3/3** | **19/22** |
| (Pfaehler et al., 2019) | **6/6** | **5/6** | **7/7** | **3/3** | **21/22** |
| (Shiri et al., 2017a) | **6/6** | **4/6** | **6/7** | **3/3** | **19/22** |
| (Varghese et al., 2019a) | **6/6** | **4/6** | **4/7** | **2/3** | **16/22** |
| (Yang et al., 2018b) | **6/6** | **4/6** | **4/7** | **3/3** | **17/22** |
| (Zhovannik et al., 2019a) | **6/6** | **5/6** | **7/7** | **3/3** | **21/22** |

**Table 5b:** Detailed information of the reported and missing items of the radiomic reporting checklist for clinical studies: Form A reports on the domain study design and phantom information; Form B on imaging, Form C on the description of the radiomic pipeline, Form D on the information about statistical analysis, and Form E on the provided information of data and metadata availability

| **Form A – Study and Phantom info** | | | | | | | | |
| --- | --- | --- | --- | --- | --- | --- | --- | --- |
| Reference | E.1  # of Scans | E.2  Data  Collection | E.3  Data  Availability | E.4  Study  Design | E.5  Phantom  Characteristics |  |  | **Reported**  **Items** |
| (Baeßler et al., 2018) |  |  |  |  |  |  |  | **6/6** |
| (Ger et al., 2020) |  |  |  |  |  |  |  | **4/6** |
| (Johnson et al., 2017) |  |  |  |  |  |  |  | **6/6** |
| (Midya et al., 2018) |  |  |  |  |  |  |  | **6/6** |
| (Nardone et al., 2020) |  |  |  |  |  |  |  | **6/6** |
| (Papp et al., 2019) |  |  |  |  |  |  |  | **6/6** |
| (Pfaehler et al., 2019) |  |  |  |  |  |  |  | **6/6** |
| (Shiri et al., 2017a) |  |  |  |  |  |  |  | **6/6** |
| (Varghese et al., 2019a) |  |  |  |  |  |  |  | **6/6** |
| (Yang et al., 2018b) |  |  |  |  |  |  |  | **6/6** |
| (Zhovannik et al., 2019a) |  |  |  |  |  |  |  | **6/6** |
|  | | | | | | | | |
| **Form B – Image acquisition and ROI(s) info** | | | | | | | | |
| Reference | F.1  Modality | F.2  Acquisition  Settings | F.3  Considered  ROI(s) | F.4  Delineation  Details | F.5  Binary Mask  Details | F.6  Pre-processing  Details |  | **Reported**  **Items** |
| (Baeßler et al., 2018) |  |  |  |  |  |  |  | **6/6** |
| (Ger et al., 2020) |  |  |  |  |  |  |  | **5/6** |
| (Johnson et al., 2017) |  |  |  |  |  |  |  | **4/6** |
| (Midya et al., 2018) |  |  |  |  |  |  |  | **6/6** |
| (Nardone et al., 2020) |  |  |  |  |  |  |  | **5/6** |
| (Papp et al., 2019) |  |  |  |  |  |  |  | **5/6** |
| (Pfaehler et al., 2019) |  |  |  |  |  |  |  | **5/6** |
| (Shiri et al., 2017a) |  |  |  |  |  |  |  | **4/6** |
| (Varghese et al., 2019a) |  |  |  |  |  |  |  | **4/6** |
| (Yang et al., 2018b) |  |  |  |  |  |  |  | **4/6** |
| (Zhovannik et al., 2019a) |  |  |  |  |  |  |  | **5/6** |
|  | | | | | | | | |
| **Form C – Software and Feature info** | | | | | | | | |
| Reference | G.1  Software  Name | G.2  Software  Availability | G.3  Programming  Language | G.4  Feature  Classes | G.5  Number of  Features | G.6  Feature  Description | G.7  Feature  Specific  Parameters | **Reported**  **Items** |
| (Baeßler et al., 2018) |  |  |  |  |  |  |  | **6/7** |
| (Ger et al., 2020) |  |  |  |  |  |  |  | **6/7** |
| (Johnson et al., 2017) |  |  |  |  |  |  |  | **3/7** |
| (Midya et al., 2018) |  |  |  |  |  |  |  | **6/7** |
| (Nardone et al., 2020) |  |  |  |  |  |  |  | **6/7** |
| (Papp et al., 2019) |  |  |  |  |  |  |  | **4/7** |
| (Pfaehler et al., 2019) |  |  |  |  |  |  |  | **7/7** |
| (Shiri et al., 2017a) |  |  |  |  |  |  |  | **6/7** |
| (Varghese et al., 2019a) |  |  |  |  |  |  |  | **4/7** |
| (Yang et al., 2018b) |  |  |  |  |  |  |  | **4/7** |
| (Zhovannik et al., 2019a) |  |  |  |  |  |  |  | **7/7** |
|  | | | | | | | | |
| **Form D – Statistical Analysis Info** | | | | | | | | |
| Reference | H.1  Metric | H.2  Cut-off(s) | H.3  Results  Availability |  |  |  |  | **Reported**  **Items** |
| (Baeßler et al., 2018) |  |  |  |  |  |  |  | **3/3** |
| (Ger et al., 2020) |  |  |  |  |  |  |  | **2/3** |
| (Johnson et al., 2017) |  |  |  |  |  |  |  | **1/3** |
| (Midya et al., 2018) |  |  |  |  |  |  |  | **3/3** |
| (Nardone et al., 2020) |  |  |  |  |  |  |  | **3/3** |
| (Papp et al., 2019) |  |  |  |  |  |  |  | **3/3** |
| (Pfaehler et al., 2019) |  |  |  |  |  |  |  | **3/3** |
| (Shiri et al., 2017a) |  |  |  |  |  |  |  | **3/3** |
| (Varghese et al., 2019a) |  |  |  |  |  |  |  | **2/3** |
| (Yang et al., 2018b) |  |  |  |  |  |  |  | **3/3** |
| (Zhovannik et al., 2019a) |  |  |  |  |  |  |  | **3/3** |

1. **Aims of the review**

- to compare the findings from recently published papers with respect to the major results found in our previous review
- to verify if there has been a boost of consensus regarding a list of major factors impacting reproducibility and repeatability
- to isolate a set of repeatable and reproducible features across different modalities in both human and phantom data.
- to verify if one of the largest issues identified in our previous work, being the poor quality of reporting, has been addressed in new studies.

1. **Eligibility criteria**

The articles included in this review needed to fulfill the following criteria:

- Publishing date between 01-01-2017 to 01-12-2020.
- Peer-reviewed full-text articles containing one of the search keywords in their title or abstract in the PubMed and Web of Science electronic databases:
  - cancer
  - radiomics (including texture analysis)
  - reproducibility
  - repeatability
- Only studies investigating radiomic features extracted A) from one of the imaging modalities CT, PET, or MRI and B) from radiologic phantoms or from human persons suffering from at least one primary tumor were included in the review.
- All included articles had to report on the repeatability/reproducibility of radiomic features to at least one of the following aspects: image acquisition or reconstruction parameters, imaging modalities, effect of image pre-processing such as image smoothing, or effect of segmentation results.
- All studies included a statistical analysis assessing the degree of robustness were included in this review.
- Only articles written in English were considered for the review.

1. **Search string**

The search string to find the articles potentially included in this review is the following:

(((((((((((((interclass[Text Word]) OR correlation[Text Word]) OR agreement[Text Word]) OR concordance[Text Word]) OR correlation[Text Word]) OR pearson[Text Word]) OR spearman[Text Word]) OR reproducibility[Text Word]) OR variation[Text Word]) OR variability[Text Word])) AND ((((((((Predict*[tiab] OR Predictive value of tests[mh] OR Scor*[tiab] OR Observ*[tiab] OR Observer variation[mh]))) OR (stratification OR roc OR discrimination OR discriminate OR c statistic OR c statistic OR area under the curve OR auc OR calibration OR indices OR algorithm OR multivariable))) OR ((((validate OR predict$.ti. OR rule$) OR (predict$ AND (outcome$ OR risk$ OR model$)) OR ((history OR variable$ OR criteria OR scor$ OR characteristic$ OR finding$ OR factor$) AND (predict$ OR model$ OR decision$ OR identify OR prognose)) OR (decision$ AND (model$ OR clinical$ OR logistic models/)) OR (prognostic AND (history OR variable$ OR criteria OR scor$ OR characteristic$ OR finding$ OR factor$ OR model$)))) OR (stratification OR roc OR discrimination OR discriminate OR c statistic OR c statistic OR area under the curve OR auc OR calibration OR indices OR algorithm OR multivariable)))) AND ((((((((radiomic*[Text Word]) OR radiogenomic*[Text Word]) OR phenotyp*[Text Word]) OR radiophenotyp*[Text Word]) OR textur*[Text Word])) AND ((("computed tomography") OR "magnetic resonance") OR "positron emission")) AND cancer[MeSH Major Topic])))) AND (("2017/05/01"[Date - Publication] : "2021/12/01"[Date - Publication]))) AND (Journal Article[Publication Type])

1. **Example for the checklist**

# In this study, 26 synthetic images were generated using a PET simulation tool. The authors mentioned all details about the phantom they used to acquire these images (signaling question A1) and the number of scans/synthetic images they acquired (question A2). However, they failed to mention the scanner at their local institution (question A3). Therefore, 1 out of 3 questions was answered with a “no” what leads to 33% ($=\frac{nr. of \text{no}s}{nr. of questions} *100\%=\frac{1}{3}*100\%$) risk of bias.

# Moreover, the authors mention clearly that they analyzed PET images in their study (question B1), they mention the image acquisition settings they simulated (question B2), and they explain the way the volume of interests were segmented (question B3). However, the study does not describe how they generated the segmentation mask from the generated contours. This leads to a risk of bias for this domain of 25%.

# Regarding the information describing the radiomic pipeline, the authors failed to report on the software they used for feature calculation (question C2), the feature aggregation as well as the feature quantization algorithm (question C5 and C6, respectively). While the authors clearly describe the image pre-processing (question C1), the radiomic feature classes and the formulas of the features (C3), as well as the number of extracted features (C4). This leads to three out of six questions, that cannot be reproduced and need therefore to be answered with a “no”. The risk of bias is therefore 50% for this group of signaling questions.

# Both questions in the group of ‘statistical analysis’ could be answered with a “yes”: The authors mention clearly the metrics used to assess features reproducibility as well as the reference standards to define the different levels of reproducibility. This leads to 0% of bias for this group.

# However, neither images nor software was shared as public data (question E1 and E2), while the results of the statistical analysis was shared in detail (question E3), i.e. the manuscript contained a list of all features including their repeatability value. This leads to a risk of bias for this feature group of 66%.

1. **Results according to disease site**

5.1 Lung

All but one PET study evaluated the reproducibility of radiomic features with respect to variability in contouring comparing manual and semi-automated algorithms. In general, all studies confirmed that the robustness is feature dependent. However, it is difficult to draw a general conclusion as all studies investigated different features. In general, most studies reported that a large number of the features were stable to delineation differences. Global textural features such as GLSZM features were more affected by differences in delineation than local textural features such as GLCM and GLRM features. However, in one PET study [23], the shape metric sphericity was found to change its prognostic value when using different delineations of the tumor. Similar results were found in CT studies, where contours delineated by different clinicians impacted the prognostic value of radiomic features. Lee et al. [28] identified nine features related to lung nodule state that were robust against differences in discretization settings and voxel size. These features included two shape, two intensity histogram based, three GLCM, and two features calculated after using additional filtering.

For CT, one study demonstrated that motion blurring and signal-to-noise ratio had an impact on radiomic feature values. However, also here the impact was feature dependent. Statistical features were found to be more robust than textural features [27]. The authors in [29,30] demonstrated that slice-thickness has a high impact on feature reproducibility. Park et al. demonstrated that the reproducibility can be improved by using a CNN-based super resolution algorithm, while Erdal et al. pointed out that a slice thickness of 2 mm leads to the most accurate shape features. However, they underlined that for textural features a standardized image acquisition protocol is essential in order to compare feature values from different centers.

5.2 Cervical cancers

One MR and two PET studies investigated the robustness of radiomic features for cervical cancers . The MR study reported that around 90% of the features were stable with respect to contouring on T2 sequences. Which is in line with a MR study of the previous review were features extracted from apparent diffusion coefficient were found to be robust against different segmentation results [31]. One PET study [32] showed that overall tumor heterogeneity patterns were profoundly affected by grey-level discretization method. GLRLM features where more robust than global textural features . In the second PET study, Whybra et al. explored the robustness and the predictive values of PET radiomic features to image interpolation to different voxel sizes and for two different interpolation algorithm [33]. Shape and statistical features were the most robust to differences in interpolated voxel sizes, while less textural features were found to be robust. The interpolation method had a high impact on feature values but not on the predictive power of the features.

5.3 Head and neck cancers

Only two PET and one MR studies investigated the reproducibility of radiomic features in head and neck cancers. In the first PET study [34], large differences were found when considering reproducibility with respect to manual, semi-automated and automated delineation methods. Overall, semi-automated and automated algorithms produced more stable results. This is in line to the findings of the previous review where a study demonstrated that a large number of feature values was robust to segmentation differences [35]. The second PET study showed strong dependencies of radiomic features with respect to digital image pre-processing parameters, but these differences were not important enough to affect the prognostic power of radiomic features. Same conclusion holds in the study about nasopharyngeal carcinoma [36].

Zhang et al. [37] explored the effects of segmentation variability on MR based radiomic feature values and found that the majority of feature values are sensitive to differences in segmentations. The differences in delineations also lead to differences in the selected features as well as their predictive value.

5.4 Pelvic region malignancies

One MR study found that first order features were found to be more reproducible than texture metrics regarding digital image pre-processing and resampling techniques when extracted from ADC maps. Among texture metrics, GLSZM features were the least and GLCM features the most robust [38]. The authors showed that the use of image pre-processing including quantization methods and resampling strongly affected reproducibility in ADC maps.

In the ADC maps from prostate cancer patients, introducing anatomical-based normalization prior to feature extraction led to more repeatable features, but this result was not confirmed for T2 weighted sequences [18].

Three studies reported on renal cell carcinomas acquired with CT imaging. One study demonstrated that CT images from corticomedullary phase were found to produce a large percentage of reproducible features with respect to variability in contouring. Opposite results were found for enhanced images. Regarding the different feature categories, two [39,40] of the studies agreed that less than 40% of computed texture features were reproducible.

5.5 Other malignancies

In MR images from oesophageal cancer patients most of the features were found to be stable with respect to variability in contouring. Same results were found in T1 sequences of breast cancer patients for both fat and non-fat saturated images.

Haarburger et al. investigated the impact of contouring variability on radiomic feature values in three cancer types for CT images [19]. They found shape and first-order features to be the most robust for all three cancer types.

For hepatocellular carcinoma, one study [17] showed that contouring did not affect the repeatability of radiomic features.

One MR study investigating the repeatability of radiomic features for brain tumors demonstrated that repeatability strongly depended on the region of interest considered. Moreover, the authors demonstrated that the choice of preprocessing algorithm had a high impact on the robustness of feature values [20]. Another MR study explored the reliability of tumor delineation method and its impact on feature robustness [41]. The authors pointed out that statistical and GLCM features were found to be more robust than GLSZM features.

A CT study with patients suffering from pancreatic carcinomas as well as a CT study investigating patients with liver metastasis confirmed that the reconstruction setting had a high impact on feature values.
